# A photochromic trinuclear dysprosium(iii) single-molecule magnet with two distinct relaxation processes†

**DOI:** 10.1039/d4ra01645a

**Published:** 2024-05-02

**Authors:** Katarzyna Rogacz, Michał Magott, Sebastian Baś, Magdalena Foltyn, Michał Rams, Dawid Pinkowicz

**Affiliations:** a Faculty of Chemistry, Jagiellonian University Gronostajowa 2 30-387 Kraków Poland dawid.pinkowicz@uj.edu.pl; b Institute of Physics, Jagiellonian University Łojasiewicza 11 30-348 Kraków Poland; c Doctoral School of Exact and Natural Sciences, Jagiellonian University Łojasiewicza 11 30-348 Kraków Poland

## Abstract

Multifunctional molecules responsive to light are highly desired as components for the construction of remotely controlled nanodevices. Here we present a Dy^III^ single molecule magnet (SMM) comprising dithienylethene (dte) photochromic bridging ligands in the form of a pyridine (py) derivative: 1,2-bis((2-methyl-5-pyridyl)thie-3-yl)perfluorocyclo-pentene (dtepy). The title trinuclear compound {[Dy^III^(BHT)_3_]_3_(dtepy)_2_}·4C_5_H_12_ (1) was synthesized by combining the low-coordinate dysprosium complexes Dy^III^(BHT)_3_ (BHT = 2,6-di-*tert*-butyl-4-methylphenolate) with dtepy bridging ligands in the ‘open’ form using *n*-pentane as a completely inert solvent. The trinuclear molecule comprises two different Dy^III^ centers due to its quasi-linear geometry: a central trigonal bipyramidal Dy^III^ ion and two peripheral ones with an approximate trigonal pyramidal geometry. Thanks to that, 1 shows two types of SMM behavior which is slightly affected by the photoisomerization of the photochromic dtepy bridges. The impact of the photoisomerization on the magnetization dynamics was studied by means of alternating current (AC) magnetic susceptibility measurements for the ‘open’ and ‘closed’ forms of the molecules. The changes between the ‘open’ and ‘closed’ isomers were further investigated by IR and UV-vis spectroscopy, suggesting the co-existence of the ligand-related photochromism and single-molecule magnet behavior in 1. However, the powder X-ray diffraction studies indicate loss of structural order in the first photoisomerization step preventing in-depth studies.

## Abstract 2

The photochromic trinuclear Dy(iii) single-molecule magnet shows two distinct magnetic relaxation processes associated with two different Dy(iii) geometry types.

## Graphical abstract

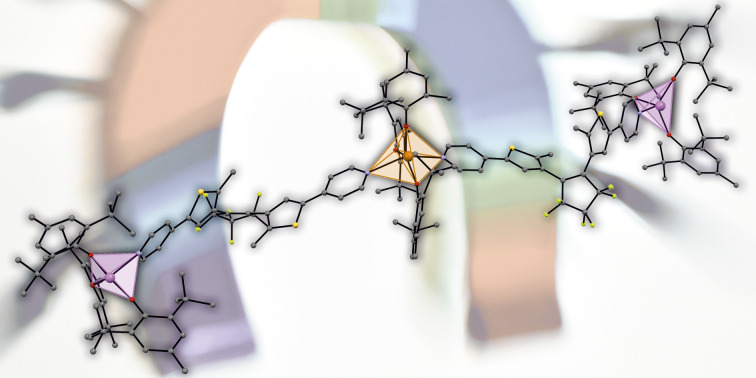

## Introduction

Multifunctional materials are being actively explored as energy storage systems,^1^ materials for life sciences,^2^ biomaterials,^3^ shape memory materials,^4^ photoactuators,^5^ sensors^6^ and flexible electronics.^7^ From the standpoint of diverse physical properties and miniaturization, multifunctional materials that combine multiple properties are most desirable. Lanthanide ions are especially attractive for the construction of multifunctional materials because they can combine many different properties such as luminescence,^8^ magnetism^9–11^ or electrochemical activity^12^ and magneto-electric effects.^13^ By employing their excited electronic states it is possible to achieve increasingly sophisticated materials with tailored properties.^14^ In the recent literature, one of the exciting applications of lanthanides is the construction of single-molecule magnets (SMMs).^15^

SMMs have received considerable attention because of their potential applications at the molecular level in data storage,^16^ quantum computing,^17^ and spintronics.^18^ In recent years, lanthanide-based single-molecule magnets (Ln-SMMs) have dominated the field. The so-called high-performance Ln-SMMs exhibit magnetization reversal barriers (*U*_eff_) exceeding 1000 cm^−1^ and record-high blocking temperatures.^19^ The best examples, dysprocenium and its derivatives, exhibit hysteresis opening above the boiling point of liquid nitrogen, demonstrating the great potential of Ln-SMMs.^20–24^

**Fig. 1:**
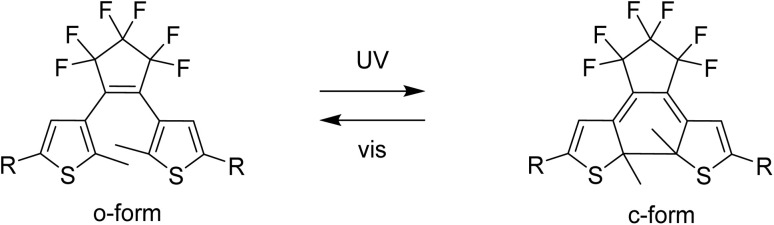
Photoisomerization of a dithienylethene-type molecule with the structural formulae of the native form o-dte (left) and the photoisomerized c-dte (right). In this work R = pyridyl.

The properties of Ln-SMM can be modified in several ways: by removing solvent molecules present in the crystal lattice or coordinated to a lanthanide ion,^25^ or by protonating the ligands.^26^ Another way to alter the properties of SMMs is to influence the magnetic state of the complex using redox-active molecules.^27–29^ A promising approach to obtain functional SMM-based devices might be the use of photoswitchable molecules.^30^ Light-responsive magnetic molecules are commonly obtained by exploiting two phenomena: light-induced excited spin state trapping (LIESST)^31,32^ or photoinduced charge transfer.^33^ An alternative strategy to achieve photo-switching is to combine a magnetic center with a photochromic molecule.^24–37^ To achieve photo-modulation of magnetic properties in lanthanide complexes, the available states of the photochromic compound must transfer magnetic interactions in different ways^38^ or affect the coordination sphere of the lanthanide ion. Photochromic dithienylethenes (dte) are a well-known family of molecular photoswitches where both isomers: the open form (o-form) and the closed form (c-form) (Fig. 1) are thermally stable.^39^ Photoisomerization of dte derivatives can occur in the picosecond timeframe^40^ with near-unity quantum yields.^41^ Furthermore, dte derivatives show high fatigue resistance during irradiation cycling^41^ and their additional advantage is the possibility of isomerization in the solid state even in a single-crystal-to-single-crystal fashion.^41^ For this to happen, dte-based compounds must meet three conditions: crystalize in an antiparallel conformation,^42^ the distance between two active carbon atoms in the thiophene rings should be less than 3.8 Å,^43^ and the photo-induced changes must be cooperative.

Herein we present the spectroscopic, magnetic and photo-switching properties of a single-molecule magnet: {[Dy^III^(BHT)_3_]_3_(dtepy)_2_}·4C_5_H_12_ (1), in which dysprosium ions are bridged by a photochromic ligand 1,2-bis((2-methyl-5-pyridyl)thie-3-yl)perfluorocyclo-pentene (dtepy) (BHT = 2,6-di-*tert*-butyl-4-methylphenolate) to form a trinuclear complex. This compound exhibits field-induced slow magnetic relaxation, but unlike the erbium analogue {[Er^III^(BHT)_3_]_3_(dtepy)_2_}·4C_5_H_12_,^44^ it shows two thermally activated relaxation processes typical for SMMs. Relaxation of the magnetization was studied for the native open form of the complex (1) and for the closed form (1-irrad) obtained after UV-light irradiation at room temperature. The photoisomerization of 1 into 1-irrad was confirmed by IR and UV-vis spectroscopy. However, only negligible effect on the magnetic properties of the Dy^III^ centers was observed, which is most probably caused by a relatively low photo-conversion of the dtepy ligands to the closed form upon irradiation of 1.

## Result and discussion

### Synthesis and crystal structure

**Fig. 2:**
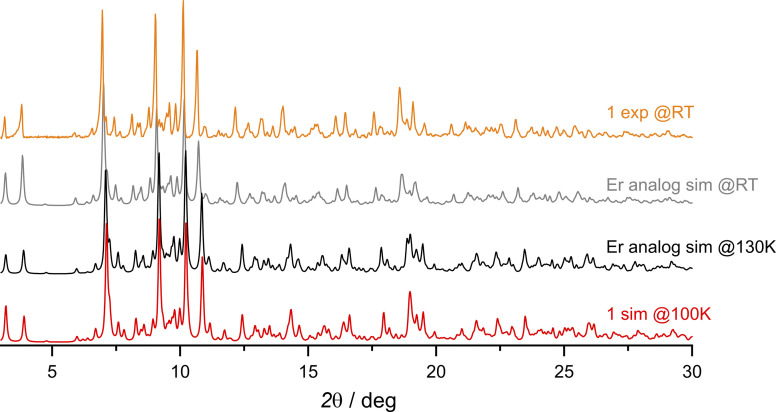
Comparison of the experimental PXRD patterns collected at room temperature (RT) for compound 1 (orange line) with the simulated ones from the scXRD data of the Er analog {[Er^III^(BHT)_3_]_3_(dtepy)_2_}·4C_5_H_12_ at RT and at 130 K (gray and black lines, respectively; ref. 44) and simulated from scXRD at 100 K for compound 1 (red line).

The synthetic procedure leading to {[Dy^III^(BHT)_3_]_3_(dtepy)_2_}·4C_5_H_12_ (1) is similar to that previously described for {[Er^III^(BHT)_3_]_3_(dtepy)_2_}·4C_5_H_12_ (Fig. S1†).^44^ The reaction takes place in a strictly non-coordinating solvent – *n*-pentane – between two equivalents of the low-coordinate Dy(BHT)_3_ substrate^46^ and one equivalent of the dtepy ligand. The result is a trinuclear linear complex in which the dtepy acts as a molecular bridge linking Dy^III^ centers. The compound crystallizes spontaneously from a yellow solution within several hours. The experimental PXRD pattern recorded for the bulk crystalline product under the mother solution at room temperature (Fig. 2, orange line) matches that simulated from the single-crystal X-ray (scXRD) diffraction structure of the Er-analog recorded at 270 K (Fig. 2; gray line). This confirms the phase purity of the investigated compound 1. Note that the magnetic studies were performed under the same conditions, for the sample immersed in the mother liquor to prevent desolvation and enable reliable structure–property correlations.

**Fig. 3:**
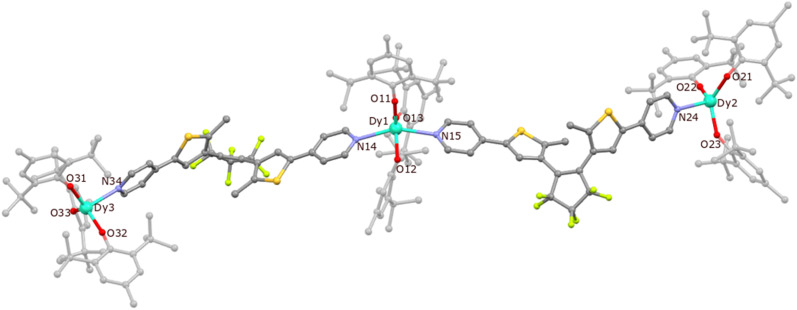
The crystal structure of {[Dy^III^(BHT)_3_]_3_(dtepy)_2_}·4C_5_H_12_ (1) with the labelling scheme. Dy – light blue, S – yellow, C – grey, N – violet, F – light-green. H atoms and *n*-pentane molecules omitted for the sake of clarity.

Compound 1 crystallizes in the monoclinic space group *P*2_1_/*c* and the dysprosium atoms are connected by dtepy acting as bridging ligands to create a trinuclear linear molecule (Fig. 3). Details of the crystal structure refinement can be found in Table S1 in the ESI.† The two peripheral dysprosium centers Dy2 and Dy3 are four-coordinated and have a distorted trigonal pyramidal (TPY) geometry (three BHT molecules are O-coordinated and one dtepy ligand is N-coordinated to the metal center). The central Dy1 is five-coordinated and has an approximate trigonal bipyramidal (TBPY) geometry (three BHT molecules are O-coordinated and occupy the equatorial positions while the two dtepy ligands are N-coordinated to the metal center and occupy the apical positions).

The crystallographic parameters for 1 are summarized in Table 1. The Dy–O bonds for the peripheral dysprosium atoms are within the 2.066(5)–2.102(5) Å range, which is similar to the previously reported complex with erbium (2.056(5)–2.094(4) Å)^44^ and other lanthanide-aryloxide complexes (within 2.035–2.144 Å range).^45–47^ The respective distances for the central dysprosium atom are slightly longer (2.096(5)–2.148(7) Å). A similar pattern can be observed for Dy–N bonds: the peripheral ones show Dy–N distances of 2.488(7)–2.501(7) Å, while the central Dy are longer (2.502(8)–2.550(7) Å). Therefore, the geometry of the peripheral Dy centers deviates from the ideal TPY geometry. This is manifested by the O–Dy–O angles which are larger than 120° (123.9(2)° for O21–Dy2–O22 and 127.1(2)° for O33–Dy3–O32). Similar distortions from the perfect TBPY are also present in the central dysprosium atom: the N–Dy–N angle is 148.2(3)° as compared to the ideal 180° and O–Dy–O angles are within 105.5(2)–136.7(2)° range while the expected value a perfect TBPY is 120° the. The shortest intermolecular distance between the Dy^III^ centers in the adjacent molecules is 10.733(1) Å, while the shortest intramolecular distance is 19.608(2) for Dy1–Dy2 and 19.362(2) for Dy1–Dy3. In both cases, these distances are not expected to influence significantly the magnetic properties of the Dy^III^ centers in 1.

**Table:** Bond lengths and angles within the coordination spheres of Dy^III^ ions in compound 1

	Central Dy*x*, *x* = 1 (Dy1)	Peripheral Dy*x*, *x* = 2 (Dy2)	Terminal Dy*x*, *x* = 3 (Dy3)
**Bond lengths/Å**			
Dy*x*–Ox1	2.096(5)	2.066(5)	2.080(5)
Dy*x*–Ox2	2.148(7)	2.102(5)	2.099(6)
Dy*x*–Ox3	2.145(7)	2.101(6)	2.096(5)
Dy*x*–Nx1	2.550(7)	2.488(7)	2.501(7)
Dy*x*–Nx2	2.502(8)		

**Angles/°**			
Ox1–Dy*x*–Ox2	112.7(2)	123.9(2)	108.3(2)
Ox1–Dy*x*–Ox3	110.6(2)	105.5(2)	118.1(2)
Ox3–Dy*x*–Ox2	136.7(2)	123.3(2)	127.1(2)
Ox1–Dy*x*–Nx4	107.1(2)	104.8(2)	109.1(2)
Ox3–Dy*x*–Nx4	84.9(3)	110.0(2)	87.1(2)
Ox2–Dy*x*–Nx4	84.5(2)	84.4(2)	100.8(2)
Ox1–Dy*x*–Nx5	104.6(3)		
Ox3–Dy*x*–Nx5	82.7(3)		
Ox2–Dy*x*–Nx5	84.9(3)		
Nx5–Dy*x*–Nx4	148.2(3)		

The coordination spheres of all three Dy^III^ ions in 1 was also investigated by means of Continuous Shape Measure (CShM) analysis using SHAPE software. Note that, CShMs are parameters that compare the experimental geometry relative to ideal reference polyhedra, CShM equal to 0 indicates perfect agreement with the reference geometry. The CShM values obtained for all three Dy^III^ ions in 1 are summarized in Table 2 below. Dy1 is best described by a square pyramid (SPY-5). However, the CShM analysis does not consider the type and charge of the donor atoms. Taking into consideration that the neutral N atoms are placed nearly in the opposite sites of the central ion and the negatively charged O atoms occupy the approximate equatorial plane, it's better to describe it as a trigonal bipyramid or a vacant octahedron. Please note that CShM values for both trigonal pyramidal shapes attain relatively low values confirming the above conclusion. The peripheral Dy2 and Dy3 show nearly identical 4-coordinate geometries with the lowest CShMs of 2.13 and 1.81 corresponding to the tetrahedral shape. However, since one of the donor atoms is neutral and the other three are charged, it is better to describe this geometry as a distorted trigonal pyramid – in fact the next lowest CShM values correspond to the vacant trigonal bipyramid which is fully consistent with TPY geometry.

Results of the Continuous Shape Measure analysis of all Dy^III^ centers in compound 1. In the case of Dy1 five reference polyhedra were considered as implemented in the SHAPE software: pentagon (PP-5, *D*_5h_), vacant octahedron (vOC-5, *C*_4v_), trigonal bipyramid (TBPY-5, *D*_3h_), spherical square pyramid (SPY-5, *C*_4v_) and Johnson trigonal bipyramid J12 (JTBPY-5, *D*_3h_). In the case of Dy2 and Dy3 four reference shapes were analyzed: square (SP-4, *D*_4h_), tetrahydron (T-4, *T*_d_), seesaw (SS-4, *C*_2v_) and vacant trigonal bipyramid (vTBPY-4, *C*_3v_) which corresponds to a trigonal pyramidal shape

**Table:** 

Dy1				
PP-5	vOC-5	TBPY-5	SPY-5	JTBPY-5
33.373	3.053	4.369	0.876	4.941

**Table:** 

Dy2			
SP-4	T-4	SS-4	vTBPY-4
27.546	2.130	7.643	3.510

**Table:** 

Dy3			
SP-4	T-4	SS-4	vTBPY-4
26.064	1.810	6.275	3.020

### Photochromic behavior of {[Dy^III^(BHT)_3_]_3_(dtepy)_2_}·4C_5_H_12_ (1)

The obtained complex {[Dy^III^(BHT)_3_]_3_(dtepy)_2_}·4C_5_H_12_ (1) shows pronounced photochromic properties. The crystals change color from light yellow to deep blue upon exposure to UV light (390 nm). This behavior is reversible to some extent by red light irradiation, which makes the crystals to turn yellow again. Photoisomerization and its influence on the properties of 1 was investigated by solid state UV-vis, IR spectroscopy (for IR spectra see Fig. S2 in the ESI†) and magnetic properties. To confirm that the photocyclization is reversible, the crystals were irradiated interchangeably with UV (390 nm) and visible light (650 nm). In the case of UV-vis investigations, the irradiation cycles were repeated several times to test the fatigue resistance of 1.

### UV-vis spectroscopy

**Fig. 4:**
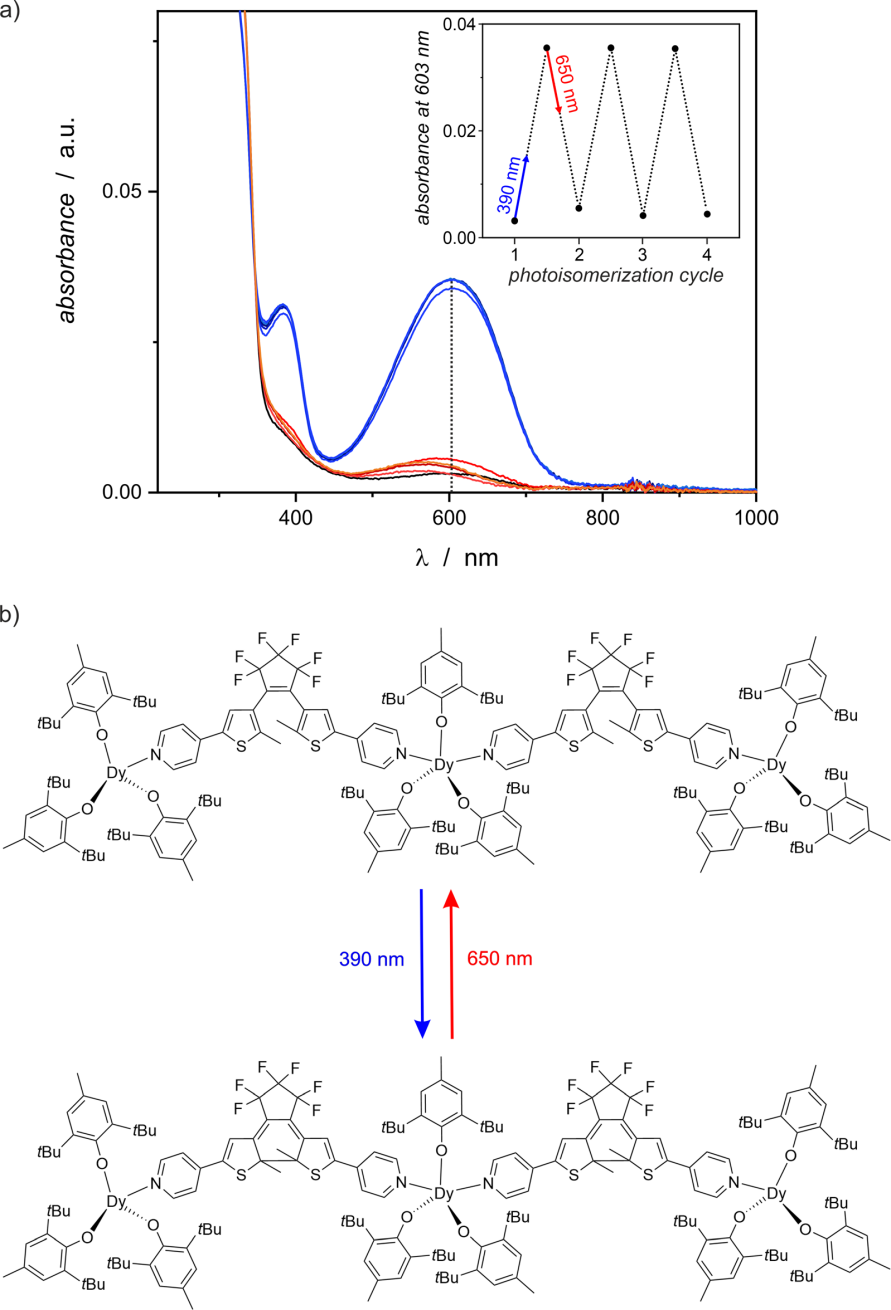
(a) UV-vis spectra showing the photochromic behavior of compound 1 in the solid state (nujol mull) at room temperature in three full photoisomerization cycles (black line = 1 before irradiation; blue and violet lines = sample after 390 nm irradiation resulting in 1-irrad; orange and red lines – sample after 650 nm irradiation leading to the restored 1). Inset: changes of the absorbance at 603 nm in three full photoisomerization cycles. (b) Diagram showing the photocyclization of dtepy ligands in compound 1 upon UV light irradiation, resulting in 1-irrad and the reverse process in response to red light.

The photochromic behavior of compound 1 was qualitatively investigated using UV-vis spectroscopy in the solid state, as shown in Fig. 4. Only weak absorption of visible light is observed for pristine 1, but 1-irrad strongly absorbs light at 600 nm. The 390 nm irradiation of the nujol mull of 1 results in a significant increase of the absorption band at 600 nm. In each photocyclization experiment the absorbance saturates around 0.036 (a.u.) after 10 minutes of irradiation, but the reverse process of 1-irrad to the open form 1 requires at least 15–20 minutes of red-light irradiation. The low absorbance at 600 nm for each 1-irrad state, indicates that the photocyclization is not complete. The photochromic switching can be repeated several times (here we demonstrated three full cycles) and each time the corresponding spectra look similar. However, slight decomposition is observed as evidenced by the absorbance around 580 nm for the open form after red-light irradiation experiments, which increases with each cycle.

### Magnetic studies

#### Direct-current (DC) magnetic properties

The magnetic properties were studied for both 1 and 1-irrad (sample of 1 in contact with mother solution, irradiated *ex situ* using 390 nm UV light). Magnetization curves for 1 and 1-irrad were measured at 2 K (Fig. S3†). At 50 kOe, the magnetization is almost saturated and reaches 15.7 μ_B_ for 1 and 15.4 μ_B_ for 1-irrad which is typical for three Dy^III^ centers (the saturation value for a single Dy^III^ center in SMMs is usually around 5.1 μ_B_ (ref. 48)).

The molar magnetic susceptibility temperature product *χT*, decreases with decreasing temperature (Fig. S3†) due to the depopulation of *m*_J_ levels, resulting from the crystal field splitting. At 100 K the *χT* value reaches 40.3 and 40.6 cm^3^ K mol^−1^ for 1 and 1-irrad, respectively. This is close to the theoretical value of 42.5 cm^3^ K mol^−1^ expected for three Dy^III^ ions in the free-ion approximation: ^6^H_15/2_, *g*_J_ = 4/3.^49^

#### Dynamic magnetic properties

AC magnetic measurements were carried out for 1 and 1-irrad. The temperature dependence of the AC magnetic susceptibility for both samples at 0 Oe is very similar (Fig. S4 and S5†), and a weak dispersion of *χ*′′ at 500 Hz is visible up to 10 K. Under applied field of 1000 Oe, on the other hand, the curves for different frequencies of the AC magnetic field are very well separated up to 20 K (Fig. S6 and S7†), and two main maxima of *χ*′′ are clearly visible indicating the presence of two main relaxation processes. The magnetic relaxations were therefore studied as temperature dependence at 1.2 kOe and 0.2 kOe, and as field-dependence at 2 K for both before and after irradiation.

**Fig. 5:**
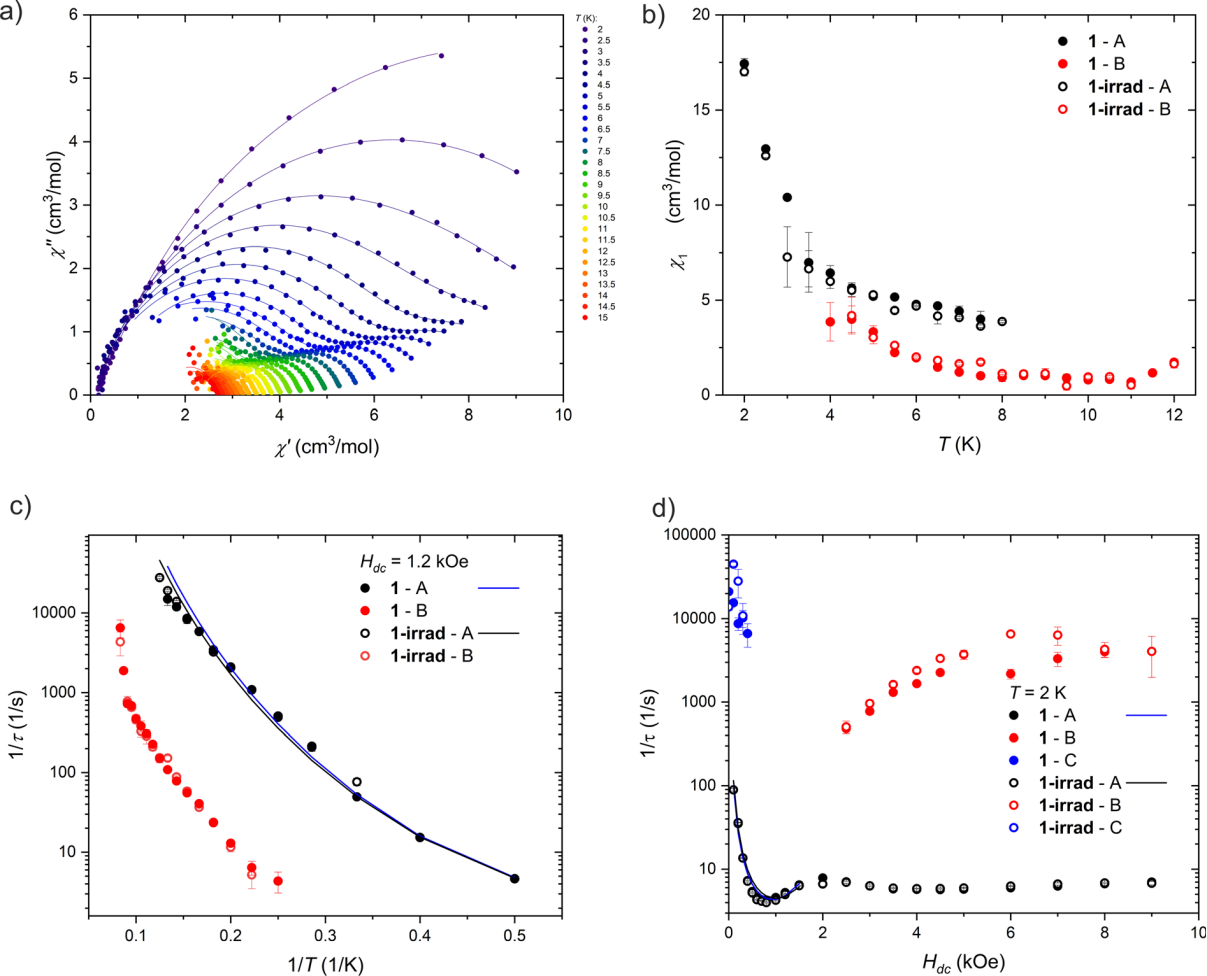
Different relaxation processes in 1 and 1-irrad. (a) Argand plot of the AC magnetic susceptibility measured at *H*_dc_ = 1.2 kOe and *H*_ac_ = 3 Oe for 1. The lines are the fits of the single or double mode Cole–Cole model. (b) Temperature dependence of the amplitude, *χ*_1_, determined from the Cole–Cole fits of the AC magnetic susceptibility measured at 1.2 kOe. (c) Temperature dependence of the magnetic relaxation times at 1.2 kOe presented as 1/*τ vs.* 1/*T*. The lines are the fits of eqn (3). (d) Field dependence of the magnetic relaxation times at 2 K presented as 1/*τ vs. H*. The lines are the fits of eqn (3).

The frequency dependence of the AC magnetic susceptibility was analyzed using the single (eqn (1)) or double (eqn (2)) mode Cole–Cole model:1
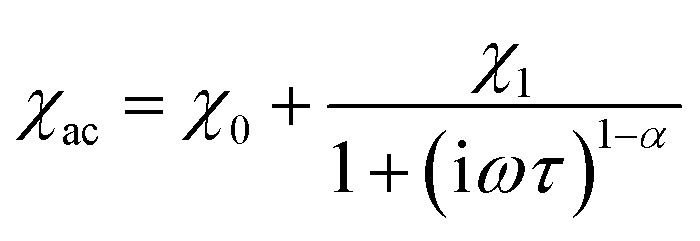
2
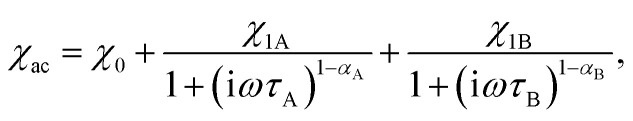
where *χ*_0_ is the adiabatic limit of the magnetic susceptibility, *χ*_1A_ and *χ*_1B_ are the magnetic susceptibilities associated with the two processes A nad B, *τ* denotes relaxation times and *α* describes the dispersion of the relaxation times. The relevant frequency dependencies of the AC magnetic susceptibility and Argand plots are presented in Fig. S8–S19.† The temperature and field dependencies of the relaxation times are presented in Fig. 5.

We were able to distinguish two main magnetic relaxation processes for compound 1: A and B, and an additional one, present only at very low field and low temperatures: C. Processes A and B can be attributed to the relaxation of the two respective Dy(iii) centers in the structure of compound 1 based on the respective values of the *χ*_1A_ and *χ*_1B_ from eqn (2). The value of *χ*_1A_ (associated with the relaxation of two terminal four-coordinated Dy(iii) ions) is always greater than the value of *χ*_1B_ (corresponding to the five-coordinate Dy(iii) ion in the middle of the molecule), as demonstrated in Fig. 5 and S20.† The *χ*_1A_/*χ*_1B_ ratio is close to 2 : 1 at both DC magnetic fields: 1.2 kOe and 0.2 kOe. This indicates that the relaxation of the five-coordinated Dy(iii) centers has a different characteristics than the relaxation of the four-coordinated centers. The process C cannot be attributed directly to a single type of Dy(iii) centers, since it is visible only at low magnetic field and very low temperature (Fig. S20†). We hypothesize that it may be an averaged signal originating from the Dy ions having their easy axis almost perpendicular to the field, and thus much weakly split ground doublet, leading to very fast relaxation even under applied magnetic field.

The relaxation times were analyzed using the following equation,^50,51^ which is the sum of the relaxation rates of Orbach, Raman, direct and quantum tunnelling of magnetization (QTM) processes, respectively:3
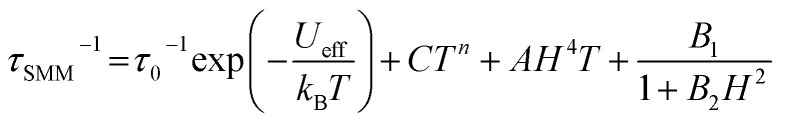


For Kramers ions with the isolated ground doublet (such as Dy(iii) in 1 and 1-irrad), the *n* parameter should be 9,^49^ however, in the case when the acoustic and optical phonons are considered, this value can vary, and thus was fitted as a free parameter.^52^

##### Process A

Process A is observed over the entire investigated temperature (2–12 K) and field (0–9 kOe) range. The inverse relaxation time (*τ*^−1^) decreases with the decreasing temperature at 1.2 kOe (Fig. 5c), which is characteristic of direct, Raman and Orbach processes. The energy barrier between the ground state and the first excited state of Dy(iii) ions in 1 is unknown, so it cannot be judged whether the Orbach mechanism contributes to the relaxation time. Moreover, this cannot be assessed from the shape of ln(*τ*) *vs. T*^−1^ relationship – the course of the points only approximates a straight line, and a similar dependence can be obtained in the limited temperature range using the Raman process.

Under increasing magnetic field (Fig. 5d), *τ*^−1^ also decreases in the range 0–0.75 kOe, and then it increases in the range 0.75–2 kOe. The initial decrease is a sign of QTM, while the increase is in turn caused by the direct process. Above 2 kOe, *τ*^−1^ decreases again, which may indicate a change of the relaxation mechanism, such as the onset of the spin-phonon bottleneck process, which suppresses direct relaxation in high DC magnetic fields. For this reason, the relaxation time was analyzed with eqn (1) only below 2 kOe.

Finally, QTM, direct and Raman mechanisms were taken into consideration in the analysis of the relaxation time of the process A (attempts to include the Orbach process failed, leading to the non-physical parameter values). Separate fitting of the relaxation time dependence (temperature-dependence for 1.2 kOe and 0.2 kOe, and field-dependence at 2 K) did not yield satisfactory results, since the *A* constant describing the direct process took a negative value in all cases. Therefore, all three sets of data were fitted simultaneously. Moreover, to limit the number of parameters, we simplified the QTM equation to *τ*_QTM_^−1^ = *B*/*H*^2^.

Such approximation is valid in this case, as the slope of *τ*^−1^(*H*) at low magnetic fields is rather steep, suggesting that already at 0.1 kOe we can assume *B*_2_*H*^2^ ≫ 1. This enables us to obtain an approximate ratio *B* = *B*_1_/*B*_2_. The simultaneous fit gives proper values of the direct, Raman and QTM processes, which are presented in Table 3. The difference between the original (1) and irradiated (1-irrad) sample is not observed, since the difference in the values of the obtained parameters is smaller than their uncertainties.

**Table:** The parameters of direct, Raman and QTM processes were obtained by fitting eqn (3) to the process A relaxation times for 1 and 1-irrad. The obtained values indicate no significant influence of the photochromic behavior on the slow magnetic relaxation in compounds 1 and 1-irrad

Parameter	1	1-irrad
*A* (kOe^−4^ K^− 1^ s^−1^)	0.36(13)	0.28(16)
*C* (K^−1^ s^−*n*^)	0.0176(53)	0.0218(73)
*n*	7.24(21)	7.00(21)
*B* (kOe^2^ s^−1^)	0.975(76)	1.124(91)

##### Process B

For process B, an insignificant field dependence is observed, but fitting all the data simultaneously (*τ*^−1^*vs. T*^−1^ and *H*) was impossible, as it led to unphysical parameters in eqn (3). Similarly to the analysis of the process A, the Orbach process was excluded from the analysis and the approximate description of QTM was used. Applying the model to only some of the data did not help obtain reasonable parameters: fitting the QTM, Raman and direct processes gives the reasonable values for the Raman process, but direct and QTM take negative values. Regardless, process B seems to be slower than process A in 1.2 kOe DC magnetic field (Fig. 5c). While the central dysprosium ion associated with the process B has two apical ligands (unlike terminal centers, with only one dtepy molecule coordinated in the axial position), the ligand field acting on dysprosium(iii) ions in 1 should rather be dominated by negatively charged BHT ligands. Therefore, the increased relaxation times of process B are most likely due to serendipitous modification of the equatorial coordination geometry of the central dysprosium. Similarly to the process A, process B does not seem to be significantly affected by the UV irradiation of 1.

##### Process C

The C process is seen only at low fields and low temperatures. This relaxation process is very fast, and its AC magnetic susceptibility is measured at the limit of the accessible frequency range. For this reason, values determined from Cole–Cole fits are subject to large uncertainties. Therefore, we did not analyze the thermal dependence of the relaxation times.

## Conclusions

The reported {[Dy^III^(BHT)_3_]_3_(dtepy)_2_}·4C_5_H_12_ (1) single molecule magnet shows a pronounced photochromic behavior associated with the reversible photoisomerization of its dithienylethene-type bridging ligand upon exposure to UV and visible light. The photochromism of 1 was confirmed by UV-vis and IR spectroscopy. Compound 1 exhibits also slow relaxation of the magnetization for both open (1) and closed (1-irrad) isomers. However, the anticipated structural changes associated with the photocyclization of the dtepy ligands have only subtle effect on the magnetic properties of the molecule. This is due to the incomplete photocyclization in the solid state as well as the remote character of these changes with respect to the ligand field of the Dy^III^ centers. This suggests that the dte-type ligands should be attached to a high-performance SMM such as dysprosocenium-^53–55^ or lanthanide-bisamide-type^56^ complexes to achieve substantial light-switchable magnetic properties. Most importantly, in the linear molecule of 1 it was possible to distinguish two processes of slow relaxation of the magnetization associated with the central and peripheral dysprosium ions. This was not observed previously for the Er^III^ analog of 1 and is a relatively rare among Ln-SMMs.^57–59^

## Conflicts of interest

There are no conflicts to declare.

## Supplementary Material

RA-014-D4RA01645A-s001

RA-014-D4RA01645A-s002
