# Supplementary material for: A photochromic trinuclear dysprosium(iii) single-molecule magnet with two distinct relaxation processes
Source: RSC Adv. 2024 May 2;14(21):14515–22. doi: 10.1039/d4ra01645a (PMC11064518; doi:10.1039/d4ra01645a)
Supplement: RA-014-D4RA01645A-s001 [file RA-014-D4RA01645A-s001.pdf]

Electronic Supplementary Material (ESI) for RSC Advances.
This journal is © The Royal Society of Chemistry 2024

Supporting Information

A Photochromic Trinuclear Dysprosium(III) Single-Molecule Magnet With Two Distinct Relaxation Processes

Katarzyna Rogacz,[1] Michał Magott,[1] Sebastian Baś,[1] Magdalena Foltyn,[2] Michał Rams[2] and Dawid Pinkowicz[1,*]

[1] Faculty of Chemistry, Jagiellonian University, Gronostajowa 2, 30-387 Kraków, Poland

[2] Institute of Physics, Jagiellonian University, Łojasiewicza 11, Kraków, 30-348, Poland

*Correspondence: dawid.pinkowicz@uj.edu.pl

Experimental section

General considerations

All reactions and sample preparations were performed under high-purity Ar gas inside the Inert PureLab HE glovebox. Solvents (HPLC) used in all syntheses were dried under Ar gas using the Inert PureSolv EN7 solvent purification system and then stored over 3 Å molecular sieves for at least 24 h prior to use. [$Dy^{III}(BHT)_3(THF)$][1] or [$Dy^{III}(BHT)_3$][2] were prepared according to literature procedures. 1,2-bis(2-methyl-5-pyridyl)thien-3-yl)perfluorocyclopentene (dtepy) ligand was synthesized according to modified literature procedures[3,4,5] starting from the commercially available substrates: 2-methylthiophene (Aldrich) and 4-iodopyridine (TCI).

Single Crystal X-ray Diffraction

Single crystal X-ray diffraction data were collected using Bruker D8 Venture equipped with the Photon III detector at 100 K (Oxford Cryostream). Mo K_α radiation generated by a microfucus sealed tube was used. Selected details of these measurements are presented in Table S1. The single crystal was taken out from the mother solution (pentane) mixed with cryoprotectant oil and mounted on the goniometers using MiTeGen cryomounts. ScXRD data were processed using Apex4 suite of programs. The structures were solved using direct methods (intrinsic phasing using SHELXT[6]) and refined anisotropically using SHELX (weighted full-matrix least-squares on F^2). Hydrogen atoms were placed in the calculated positions and refined as riding on the parent atoms. Mercury 2020.2.0 software (CCDC) was used to visualize the scXRD structural models and prepare the structural diagrams presented in the paper. CCDC 2334638 (compound **1** at 100 K), contain the supplementary crystallographic data for this paper, which can be obtained free of charge from the Cambridge Crystallographic Data Centre via: https://www.ccdc.cam.ac.uk/structures/.

Powder X-Ray Diffraction

PXRD measurements were performed using Bruker D8 Advance Eco (Cu K_α) equipped with Lynxeye silicon strip detector. A polycrystalline sample of compound **1** was loaded into a 0.5 diamater borosilicate capillary under the mother solution inside the glovebox (under strictly air-free conditions). The capillary was sealed using silicon grease and mounted on a goniometer head using beeswax. The PXRD was collected in three runs in the 3−50 deg 2θ range to verify if the sample did not decompose during the measurement. No signs of decomposition were observed. The resulting experimental PXRD is presented in Figure 2 alongside the PXRD patterns for the Er-analogue under the same conditions and the simulated pattern using scXRD data for both **1** and its Er-analog at low temperature.

UV-vis, IR and ^{1}H NMR Spectroscopy

IR spectra were measured for a nujol mull between two 1.0 mm thick BaF_2 plates using Nicolet iN10 MX FTIR microscope. UV-vis experiments were performed using a Shimadzu UV-3600i Plus spectrophotometer in a transmission mode for a sample of **1** as nujol mull pressed between two 1.0 mm thick quartz plates. The irradiation experiment (Figure S2) was performed *in situ* without removing the sample from quartz plates. 365 nm power LED (3 W) was used for photocyclization, and 638 nm power LED (3 W) was used for the reverse process. ^{1}H NMR spectra were recorded on Bruker spectrometers operating at 300 MHz in CDCl3.

Magnetic measurements

Magnetic measurements were performed using a Quantum Design Magnetic Properties Measurement System 5XL magnetometer. Polycrystalline samples were placed in glass tubes under mother solution of pentane and sealed under vaccum after freezing the content in LN_2. The measurements for **1** and **1-irrad** started at 100 K to ensure the pentane was frozen to avoid orienting the magnetic moments along the easy axis in the magnetic field. The direct-current (DC) magnetic moment was corrected for the diamagnetism of the sample and pentane. The phase-shift correction was applied for the results of the AC magnetic measurements. The correction was based on the frequency-dependent calibrations for Gd_2O_3.

Preparation of {[Dy^{III}(BHT)$_3$]$_3$(dtepy)$_2$}·4C$_5$H$_{12}$ (1)

{[Dy^{III}(BHT)$_3$]$_3$(dtepy)$_2$}·4C$_5$H$_{12}$ was synthesized similar to {[Er^{III}(BHT)$_3$]$_3$(dtepy)$_2$}·4C$_5$H$_{12}$. [Dy^{III}(BHT)$_3$] (0.046 g, 0.058 mmol) is dissolved in *n*-pentane (20.0 g) and dtepy (0.020 g, 0.038 mmol) is added in one portion. The mixture is stirred for ca. 15 minutes until it becomes clear and is left for crystallization at room temperature. Small yellow crystals form within 1-2 days. The identity and purity of the compound is confirmed by powder X-ray diffraction (PXRD) measurement (Figure S1). Yield: 40 mg (27%)

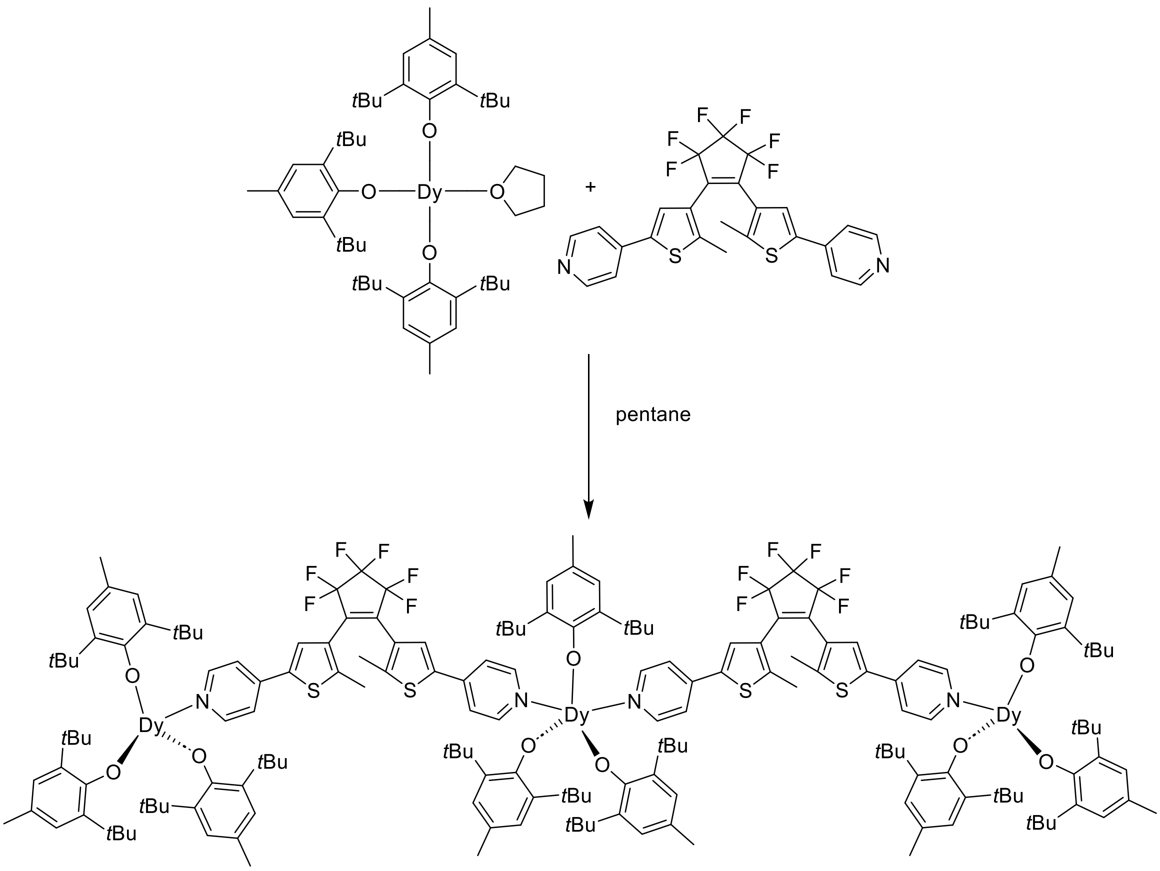

Figure S1. Schematic diagram showing the synthetic pathway to compound **1** with the structural furmulae of the substrates and the product.

IR spectra

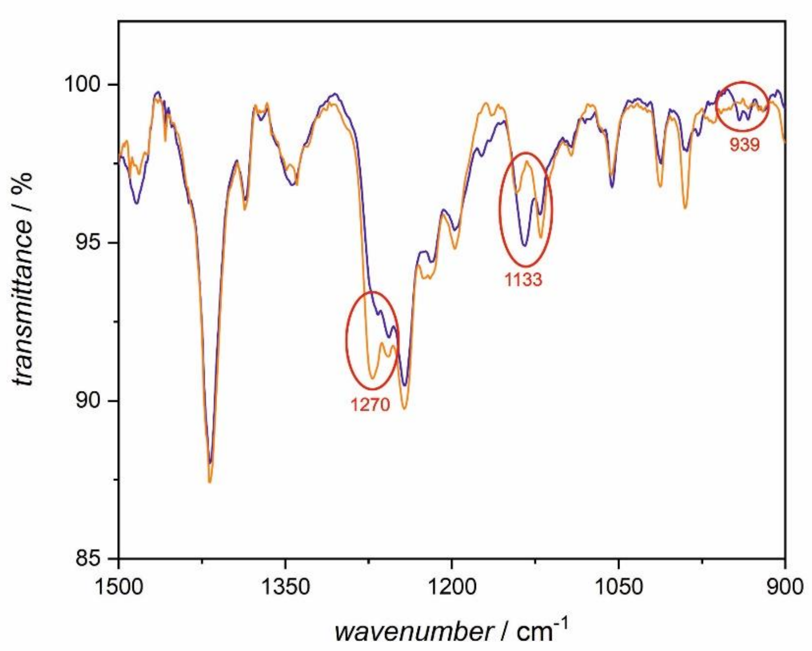

Figure S2. IR spectrum of **1** with the open form of dtepy ligand (orange line) and IR spectrum of **1-irrad** after UV light irradiation causing photocyclization of the dtepy ligand (violet line). There are three main differences: two new bands appear at 1133 cm^{-1} and 939 cm^{-1} for **1-irrad** as compared to **1**. A slight difference is also observed at 1270 cm^{-1}. All photo-induced changes are associated with the photocyclization of dtepy.

Table S1. Selected crystallographic parameters for compound **1**.

Chemical formula	$\{[Dy^{III}(BHT)_3]_3(dtepy)_2\}\cdot 4C_5H_{12}$
CCDC number	2334638
Instrument	Bruker D8 Venture
Radiation	Microfocus Mo Kα (λ = 0.71073 Å)
Formula	$C_{205}H_{287}Dy_3F_{12}N_4O_9S_4$
M_r / g mol^{-1}	3795.11
T / K	100.0
Crystal system	monoclinic
Space group	$P2_1/c$
a / Å	14.3029(5)
b / Å	24.7870(9)
c / Å	55.5667(17)
α / °	90
β / °	95.516(1)
γ / °	90
V / Å^3	19608.6(12)
Z	4
ρ_{calc} / g cm^{-3}	1.286
μ / mm^{-1}	1.241
F (000)	7948.0
Crystal size / mm^3	0.06 × 0.01 x 0.01
ϑ range / °	1.91 – 25.03 (max)
Reflections collected	117098
Independent reflections	34050
R_{int}	0.1402
Parameters/restraints	2287/306
R_1 [$F_o > 2\sigma(F_o)$]	0.0668
wR_2 (F^2)	0.1578
GOF on F^2	0.993
$\Delta\rho_{max}$, $\Delta\rho_{min}$ / e Å^{-3}	1.601 / -1.029
Completeness / %	98.4

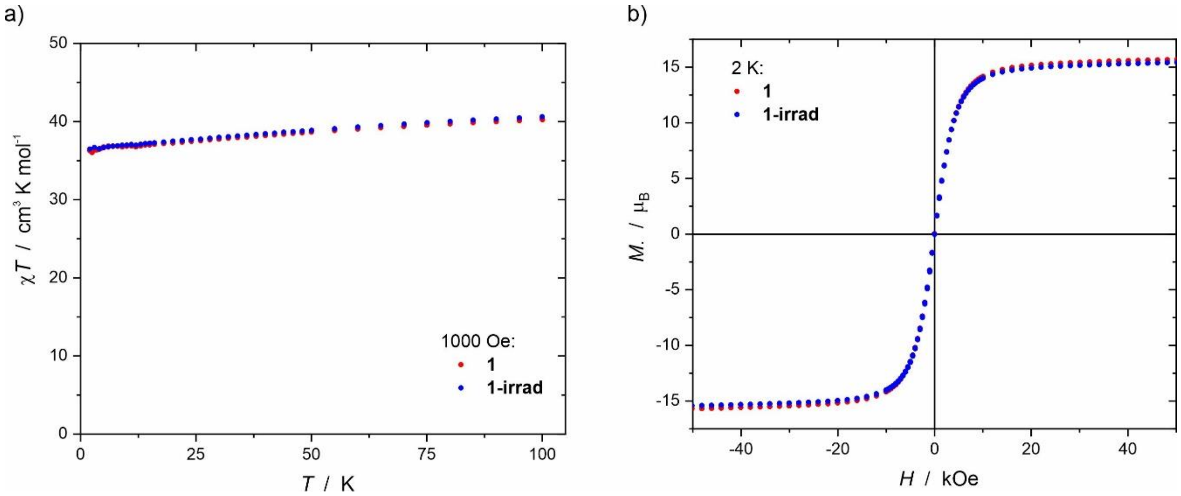

Figure S3. Temperature dependence of the molar magnetic susceptibility, χ, presented as χT product, measured for **1** and **1-irrad** (a). The measurement range was limited to 100 K in order to avoid sample alignment in the magnetic field above the metling point of *n*-pentane mother solution. Field dependence of the molar magnetization *M* for **1** (red) and **1-irrad** (blue) measured at 2 K. b)

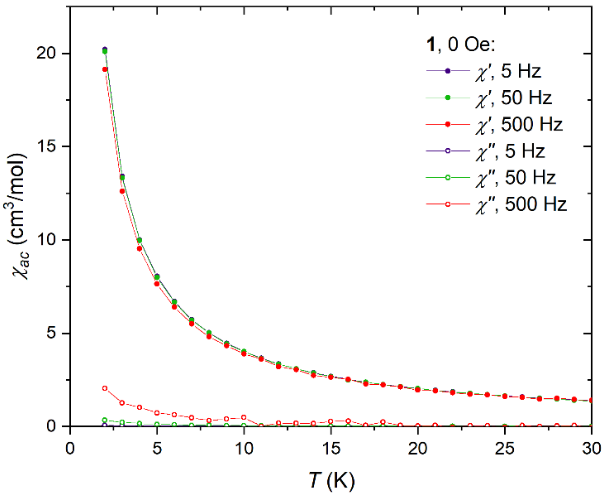

Figure S4. Temperature dependence of the AC magnetic susceptibility measured at H_{dc} = 0 Oe for **1**. The lines are the guides for the eye.

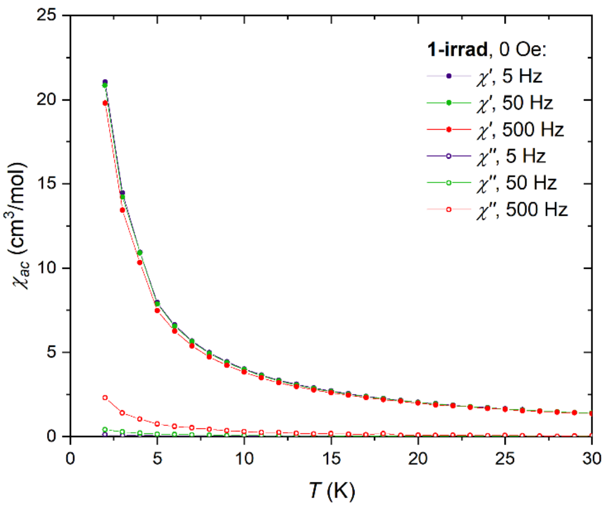

Figure S5. Temperature dependence of the AC magnetic susceptibility measured at H_{dc} = 0 Oe for **1-irrad**. The lines are the guides for the eye.

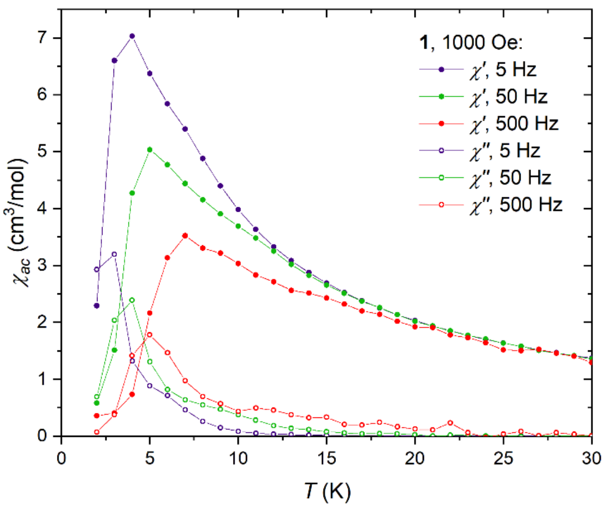

Figure S6. Temperature dependence of the AC magnetic susceptibility measured at H_{dc} = 1000 Oe for **1**. The lines are the guides for the eye.

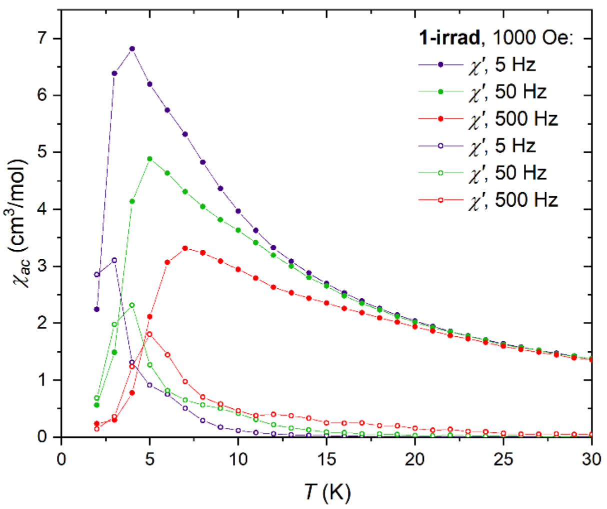

Figure S7. Temperature dependence of the AC magnetic susceptibility measured at H_{dc} = 1000 Oe for **1-irrad**. The lines are the guides for the eye.

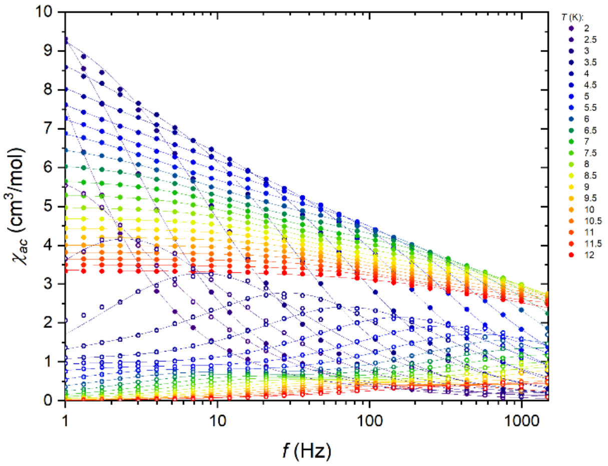

Figure S8. Frequency dependence of the AC magnetic susceptibility measured at H_{dc} = 1.2 kOe and H_{ac} = 3 Oe for **1**. The lines are the fits of the single or double mode Cole-Cole model.

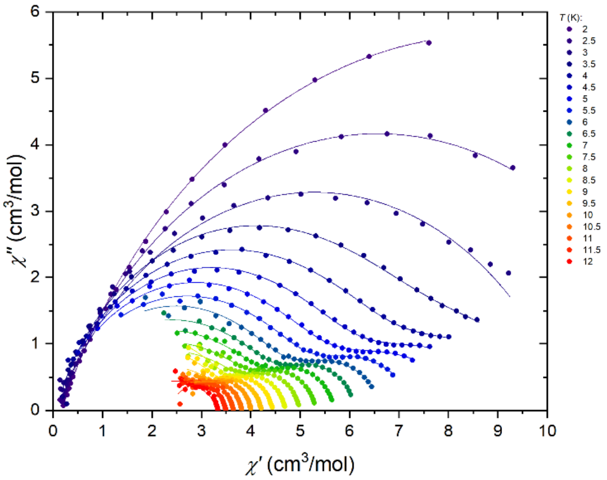

Figure S9. Argand plot of the AC magnetic susceptibility measured at H_{dc} = 1.2 kOe and H_{ac} = 3 Oe for **1**. The lines are the fits of the single or double mode Cole-Cole model.

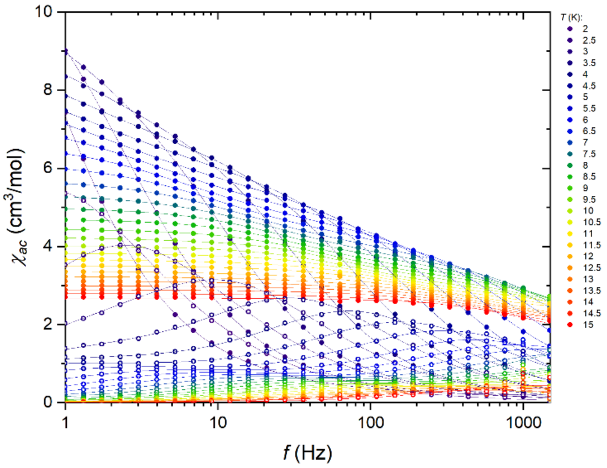

Figure S10. Frequency dependence of the AC magnetic susceptibility measured at H_{dc} = 1.2 kOe and H_{ac} = 3 Oe for **1-irrad**. The lines are the fits of the single or double mode Cole-Cole model.

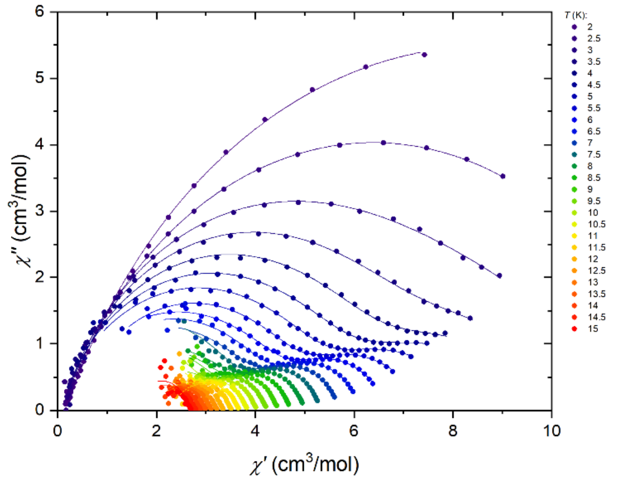

Figure S11. Argand plot of the AC magnetic susceptibility measured at H_{dc} = 1.2 kOe and H_{ac} = 3 Oe for **1-irrad**. The lines are the fits of the single or double mode Cole-Cole model.

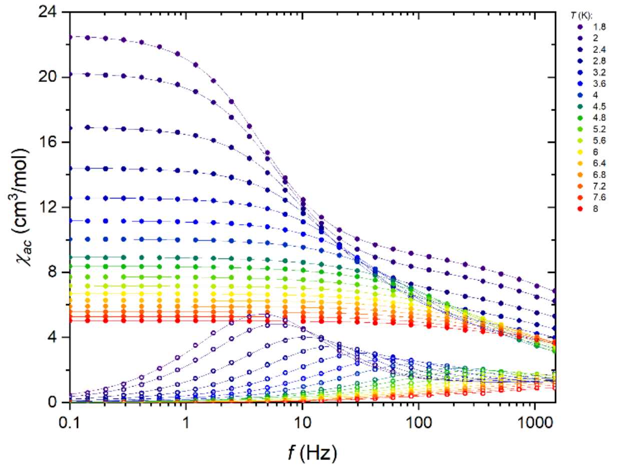

Figure S12. Frequency dependence of the AC magnetic susceptibility measured at H_{dc} = 0.2 kOe and H_{ac} = 3 Oe for **1**. The lines are the fits of the single or double mode Cole-Cole model.

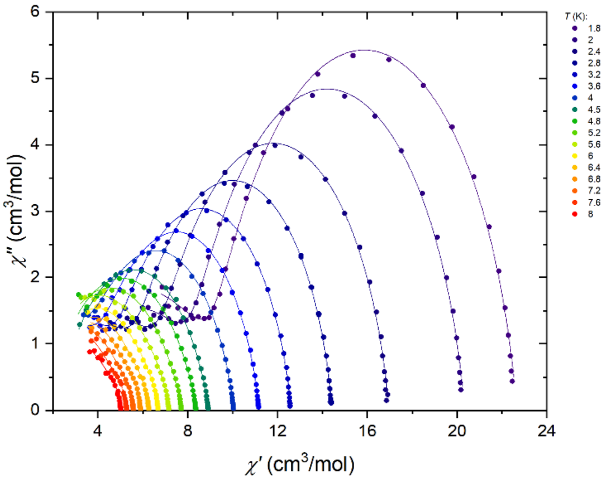

Figure S13. Argand plot of the AC magnetic susceptibility measured at H_{dc} = 0.2 kOe and H_{ac} = 3 Oe for **1**. The lines are the fits of the single or double mode Cole-Cole model.

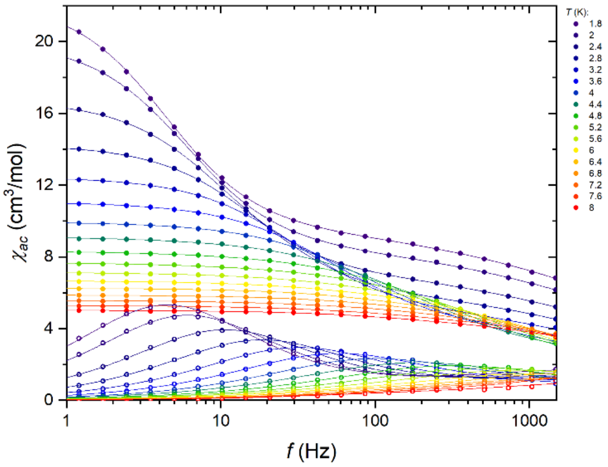

Figure S14. Frequency dependence of the AC magnetic susceptibility measured at H_{dc} = 0.2 kOe and H_{ac} = 3 Oe for **1-irrad**. The lines are the fits of the single or double mode Cole-Cole model.

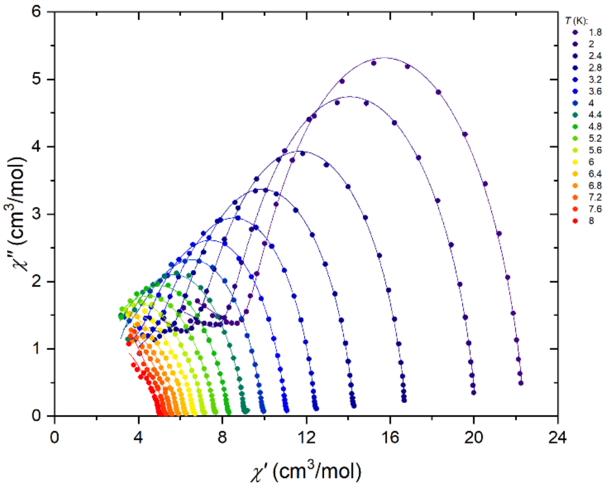

Figure S15. Argand plot of the AC magnetic susceptibility measured at H_{dc} = 0.2 kOe and H_{ac} = 3 Oe for **1-irrad**. The lines are the fits of the single or double mode Cole-Cole model.

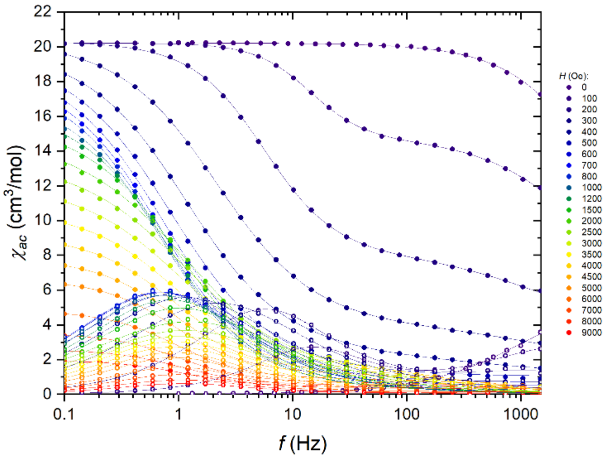

Figure S16. Frequency dependence of the AC magnetic susceptibility measured at 2 K and H_{ac} = 3 Oe for **1**. The lines are the fits of the single or double mode Cole-Cole model.

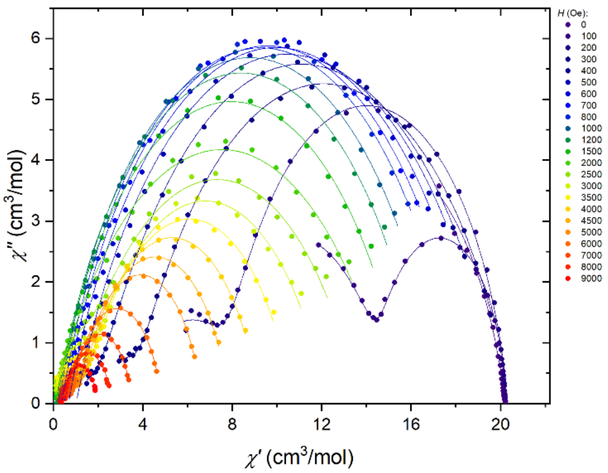

Figure S17. Argand plot of the AC magnetic susceptibility measured at 2 K and H_{ac} = 3 Oe for **1**. The lines are the fits of the single or double mode Cole-Cole model.

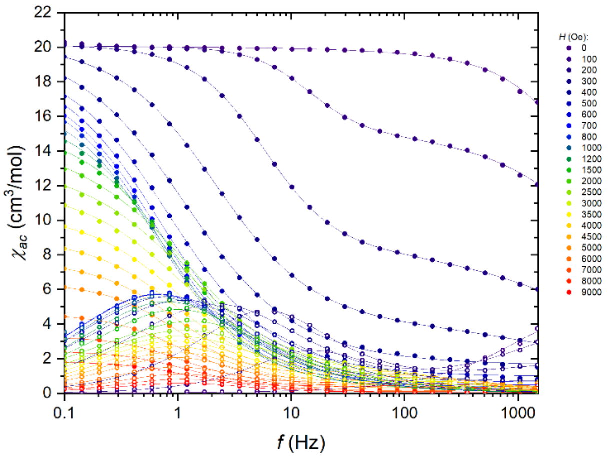

Figure S18. Frequency dependence of the AC magnetic susceptibility measured at 2 K and H_{ac} = 3 Oe for **1-irrad**. The lines are the fits of the single or double mode Cole-Cole model.

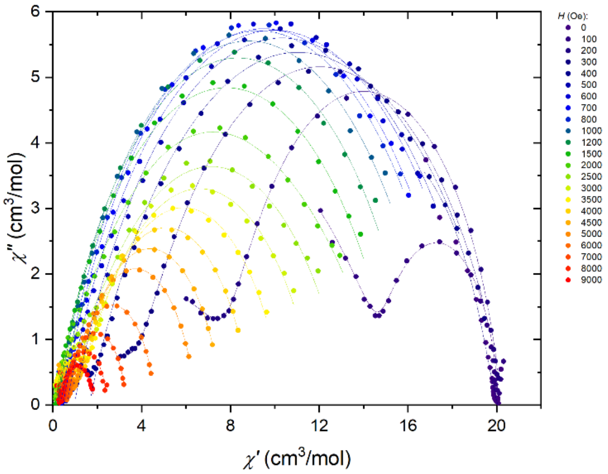

Figure S19. Argand plot of the AC magnetic susceptibility measured at 2 K and H_{ac} = 3 Oe for **1-irrad**. The lines are the fits of the single or double mode Cole-Cole model.

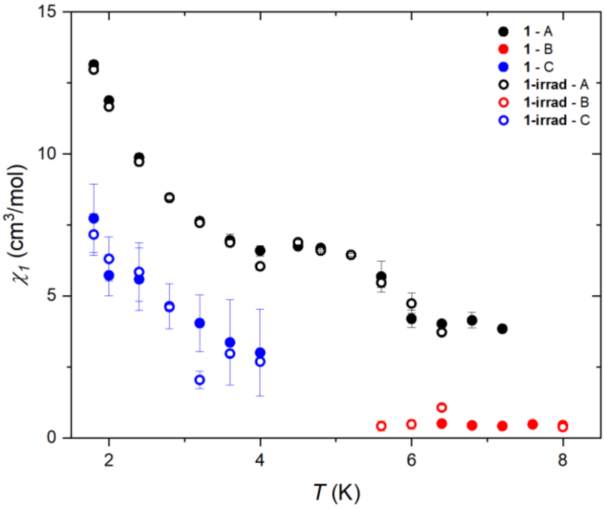

Figure S20. Temperature dependence of the χ_1 as defined in eqs 1 and 2 (main text), determined from the Cole-Cole fits of AC magnetic susceptibility measured at 0.2 kOe.

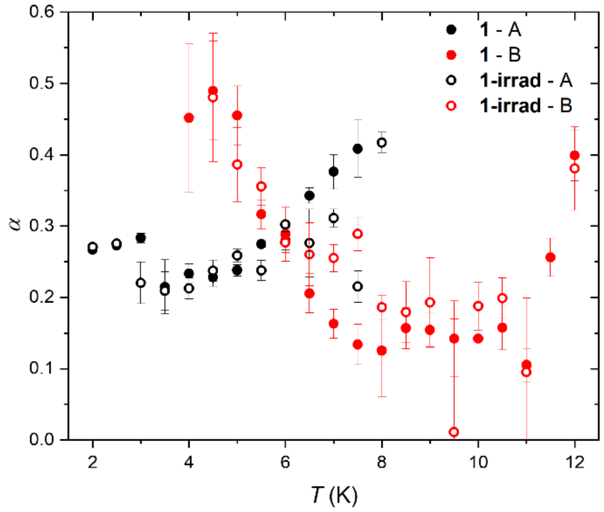

Figure S21. Temperature dependence of the α parameter determined from the Cole-Cole fits of AC magnetic susceptibility measured at 1.2 kOe.

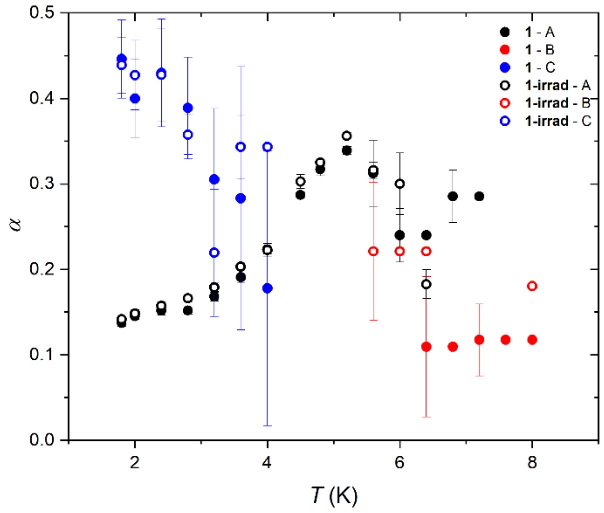

Figure S23. Temperature dependence of the α parameter determined from the Cole-Cole fits of AC magnetic susceptibility measured at 0.2 kOe.

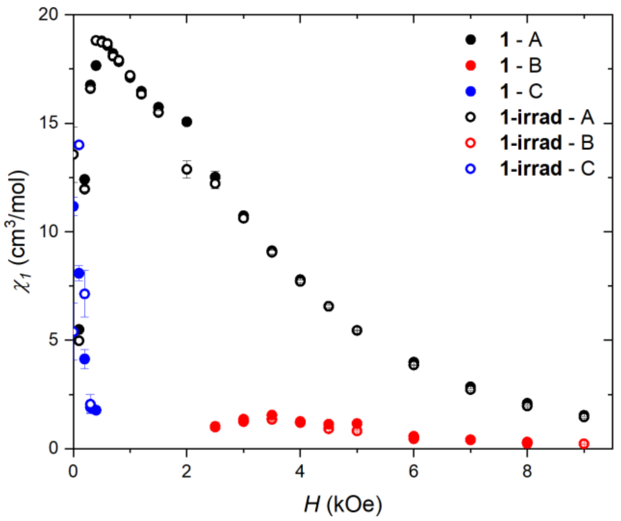

Figure S24. Field dependence of the χ_1 as defined in eqs 1 and 2, determined from the Cole-Cole fits of AC magnetic susceptibility measured at 2 K.

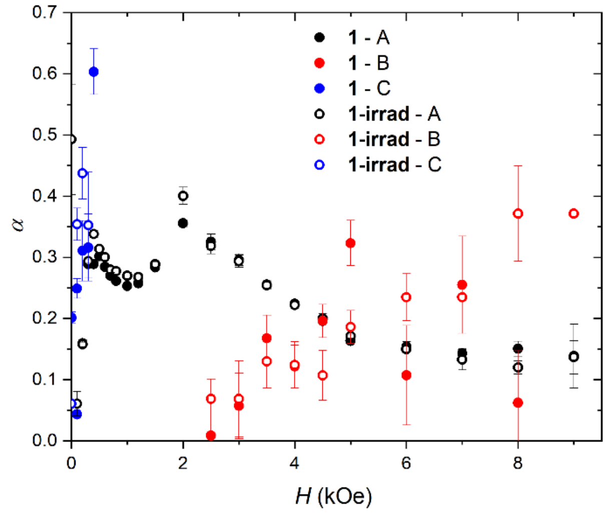

Figure S25. Field dependence of the α parameter determined from the Cole-Cole fits of AC magnetic susceptibility measured at 2 K.

References:

1. M. Brzozowska, G. Handzlik, K. Kurpiewska, M. Zychowicz, D. Pinkowicz, *Inorg. Chem. Front.* 2021, **8**, 2817.
2. H. Zhang, R. Nakanishi, K. Katoh, BK. Breedlove, Y. Kitagawa, M. Yamashita, *Dalton Trans.,* 2018, **47**, 302.
3. SH. Kawai, SL. Gilat, R. Ponsinet, J-M. Lehn, *Chem. Eur. J.,* 1995, **1**, 285.
4. V. Nikolayenko, D. Castell, P. Heerden, L. Barbour, *Angew. Chem. Int. Ed.,* 2018, **57**, 12086.
5. V. Valderrey, A. Bonasera, S. Fredrich, S. Hecht, *Angew. Chem. Int. Ed.,* 2017, **56**, 1914.
6. G. Sheldrick, *Sect. A: Found. Adv*., 2015, **71**, 3.
